# Parent-child communication about sexual issues in Zambia: a cross sectional study of adolescent girls and their parents

**DOI:** 10.1186/s12889-020-09218-y

**Published:** 2020-07-16

**Authors:** Katja Jezkova Isaksen, Patrick Musonda, Ingvild Fossgard Sandøy

**Affiliations:** 1 Plan International Norge, Tullins gate 4C, 0166 Oslo, Norway; 2 Centre for International Health, University in Bergen, Årstadveien 21, Postboks 7804, 5020 Bergen, Norway; 3 School of Public Health, University of Zambia, PO Box 50110, Lusaka, Zambia; 4 Centre for Intervention Science in Maternal and Child Health (CISMAC), Centre for International Health, University in Bergen, Årstadveien 21, Postboks 7804, 5020 Bergen, Norway

**Keywords:** Adolescents, Parents, Communication, Sexual and reproductive health

## Abstract

****B**ackground:**

Parent-child communication about sexual issues can reduce risky sexual behaviour amongst adolescents. Risky sexual behaviour is of concern in sub-Saharan Africa where the prevalence of early pregnancy, unsafe abortion and HIV are high. Parent-child communication about sexual issues presents a feasible approach for reducing sexual risk amongst adolescents in sub-Saharan Africa but limited research exists from the region. This study from Zambia examines the sociodemographic and psychosocial factors that are associated with whether parents communicate with their daughters about sexual issues.

**Methods:**

Data from a cluster randomized controlled trial examining the effect of interventions aiming to reduce teenage pregnancy and school drop out in Zambia was used. The data was collected between January–July in 2018 and consists of structured, face to face interviews with 4343 adolescent girls and 3878 parents. Cross sectional analyses examined the associations between parent-child communication about sexual issues and sociodemographic and psychosocial characteristics using univariate and multivariable logistic regression models.

**Results:**

Adolescent girls who felt connected to their parents and those who perceived their parents to be comfortable in communicating about sex, were more likely to speak to their parents about sexual issues than those who did not (AOR 1.23, 95% CI 1.01–1.52; and AOR 2.94, 95% CI 2.45–3.54, respectively). Girls whose parents used fear-based communication about sexual issues, and those who perceived their parents as being opposed to education about contraception, were less likely to communicate with their parents about sex than those who did not (AOR 0.76, 95% CI 0.65–0.89; and AOR 0.76, 95% CI 0.63–0.91, respectively). Girls enrolled in school were less likely to communicate with their parents about sex than those out of school (AOR 0.56, 95% CI 0.44–0.71).

**Conclusion:**

Parenting style, children’s perception of parental attitudes and parental communication styles are associated with whether parents and children communicate about sexual issues. This may imply that parents can improve the chances of communicating with their children about sex by conveying non-judgemental attitudes, using open communication styles with neutral messages and appearing comfortable whilst displaying positive attitudes towards communication around sex and contraceptive use.

**Trial registration:**

ISRCTN registry: ISRCTN12727868, (4 March 2016).

## Background

By communicating with their adolescents^1^ about sexual issues, parents^2^ can reduce adolescents’ risky sexual behaviours [1–3]. Examples of risky sexual behaviour amongst adolescents are early sexual debut, low condom and contraceptives use and high rates of partner change [4]; all of which increase the risk of early and unwanted pregnancy, sexually transmitted infections and HIV (Human Immunodeficiency Virus) amongst adolescents. In addition, it seems that parents can learn and improve their skills in communicating about sexual issues. This has been shown in a number of randomized controlled trials in North America where interventions aimed at improving parents’ knowledge and skills to communicate with their adolescents about sexuality issues, can significantly reduce adolescent sexual risk behaviour [5, 6].

Adolescent sexual and reproductive health (ASRH) has received growing attention in global public health due to its direct and *lasting* impact on health and well-being. Complications due to early pregnancy is amongst the leading causes of death amongst 15–19 year old girls [7] and 3.9 million girls aged 15 to 19 years undergo unsafe abortions every year due to unintended pregnancies [8]. Furthermore, early pregnancy curtails girls’ access to education and employment prospects which results in economic strains and increased risk of poverty. In sub-Saharan Africa (SSA), adolescent sexual and reproductive health is of particular concern due to the disproportionately high rates of adolescent pregnancy and HIV in the region. In 2017, 70% of new HIV infections amongst adolescents occurred in sub-Saharan Africa [9] and in 2018, the adolescent birth rate was 101 per 1000 girls [10], in comparison to a global average of 44 per 1000 [11].

The current study was set in rural Zambia. Zambian adolescents face a number of sexual and reproductive health challenges. In 2012, there were 141 births per 1000 girls between 15 and 19 [11] and adolescent childbearing rates are higher in rural than in urban areas [12]. Although maternal mortality is lower than the regional average, the pregnancy related mortality ratio among 15–19 year old girls is 80% higher than among those aged 20–24 [12]. Furthermore, the discourse around early pregnancy in rural Zambia is laden with cultural (often conflicting) beliefs, taboos and misconceptions [13].

The promising role that parent-child communication about sexual issues can play in reducing adolescent sexual risk behaviour has lead researchers and global actors to include it in their efforts to address ASRH [14]. However, whether or not parents communicate with their children about sexual issues is affected by several factors, including social and cultural norms and misperceptions around the consequences of communicating with children about sex. For example, up to 70% of American adolescents report communicating with their parents about sex [15, 16], whereas studies from African countries such as Burkina Faso, Uganda, Malawi and Ghana indicate that the percentages there are significantly lower (below 40%) [17]. Lower rates of parent-child communication about sex in African countries is often attributed to strong cultural taboos around such topics [18]. For example, secrecy and shame associated with pre-marital sex prevents parents from initiating communication about sexual issues and many parents are concerned that sharing information about sexual issues will encourage sexual initiation [19–23].

Despite more barriers and lower prevalence of parent-child communication about sex in SSA, there are indications that it is also associated with reduced risky adolescent sexual behaviour in this region [19]. For example, nascent evidence suggests that parent-child communication has been associated with greater condom use in Uganda [17], South Africa and Tanzania [24] and in Ivory Coast, parent-child communication about sexual issues was associated with a reduction of sexual partners amongst adolescents [25]. A systematic review of studies in SSA also concluded that a lack of parental guidance and communication about sexual issues is in fact a determinant of adolescent pregnancy in the region [26]. In addition to this there seems to be a desire amongst both adolescents and parents to use parent-child communication in addressing the high burden of sexual and reproductive ill health in the region [19, 20, 22, 27, 28]. For example, amongst other studies, Nigerian mothers showed strong beliefs that parents should be involved in their child’s sexuality education [29] and in Tanzania, adolescents have suggested that parent-child communication about sexual issues may be an effective means of reducing teenage pregnancies [30].

In order to increase parent-child communication about sexual issues and capitalize on its protective effects, it is first necessary to understand the specific factors that enable and encourage it. Empirical studies, largely from the ‘global north’, suggest that both sociodemographic and psychosocial factors are associated with whether or not parents and children communicate about sexual issues. Communication appears to be more common between female parents and girl children and increases with the age of the child [20, 27, 31–35]. Parents’ education and socio-economic status however have not been consistently associated with communication about sex; in North-American samples the results have been mixed but in a number of African countries higher socio-economic status [27] and higher levels of education amongst parents have been positively associated with communication about sex [29]. Parents with conservative attitudes towards adolescent sex, are less likely to communicate with their children about sexual issues [15, 36, 37] and if they do, studies in Tanzania, South Africa and Ghana have found that they may be more likely to address topics around puberty and abstinence, as opposed to condoms and contraceptives [24, 38].

In a number of North American studies, parents’ communication style and the way they formulate their messages have been shown to influence the occurrence of parent-child communication about sexual issues. Topics around sex and sexuality are inherently private, embarrassing and uncomfortable to talk about and thus not surprisingly, parental discomfort is one of the most widely cited barriers to parent-child communication about sexual issues [39, 40]. If parents feel uncomfortable, it has direct consequences for how their messages are perceived by children and thus their willingness to approach them about such issues; “By appearing extremely uncomfortable or unable to discuss sex-related topics, parents send a message to their daughters that sex is difficult to discuss, secretive, or dirty” [41] (pg. 47). Similarly, parental communication which is one-directional, instructive and uses fear-based messaging to prevent sexual activity, is likely to prevent communication about sex and may also result in overall rejection of the message being communicated [42, 43]. Parent-child connectedness has also been strongly associated both with parent-child communication and reduced risk behaviour and reduced *sexual* risk behaviour amongst adolescents [44–47] in North American studies. Connectedness is a key feature of the parent-child relationship and refers to a child’s perception of her parents as caring, interested and responsive [48]; by communicating with their children about particularly sensitive topics such as sexual issues, parents show that they care for and are concerned for their children. Although a number of qualitative studies suggest that these factors may influence parent-child communication in sub-Saharan Africa [18, 19, 49–51], no quantitative studies have hereto confirmed these associations in the region.

### The present study

Parent-child communication about sexual issues has the potential to bolster existing efforts to improve adolescent sexual and reproductive health in SSA. As noted, although some studies on the determinants of parent-child communication about sexual issues have been conducted, there remains a “dearth of evidence from SSA” which quantitatively examines the specific characteristics associated with parent-child communication around sexual issues [20] (pg. 15). Specifically, there is a lack of studies from the region which empirically examine the specific factors which can increase or reduce the likelihood that parents and children communicate about sexual issues. Therefore, this study is set in rural Zambia and aims to identify the specific sociodemographic and psychosocial factors that are associated with whether parents communicate with their children about sexual issues. Given the reportedly low rates of parent-child communication about sexual issues in SSA, it is hoped that the results will contribute to the development of evidence-based approaches which include parents in ASRH programming.

## Methods

### Study context

This study is a cross sectional study using data from a cluster randomized controlled trial. The Research Initiative to Support the Empowerment of Girls (RISE) trial was a collaboration between the University of Bergen, Norway and the University of Zambia (UNZA) which investigated the prevention of early pregnancy, school dropout and early child marriage amongst adolescent girls in Zambia. The RISE trial had three study arms: 1) intervention with economic support, 2) intervention with economic support combined with youth clubs and parent/community dialogue meetings with sensitization on adolescent sexual and reproductive health, and 3) control. In 2016, a total of 4922 girls enrolled in grade 7 were recruited from 157 rural schools in 12 districts in the Southern and Central provinces of Zambia. According to unpublished Zambian census data, the chosen districts had medium rates of school drop-out and adolescent marriage and childbearing were common [52]. All girls enrolled in grade 7 were eligible for the study, including those who were married and/or with children.^3^

### Participants and data

The data for this article are based on the data the 4th follow-up interview of the RISE study only. The 4th follow-up took place between January–July 2018 when the intervention had been implemented for approximately 1.5 years. The parents/guardians of the participants were also interviewed in this round, using structured, face to face interviews.^4^ The majority of the interviews were conducted on the school premises but in cases where the participant could not come to the school, they were interviewed in or near their homes. The questionnaires used for the 4th follow-up (girls’ and parents’ interviews) were developed together by the Zambian and Norwegian research team (see Additional file 1). The 4th interview round included questions relating to the girls’ experiences of communicating with their parents/guardians about sexual issues and their perceptions of their parents’ attitudes. The interviews with parents/guardians collected sociodemographic information and included questions on their attitudes towards adolescent sexual and reproductive health, sexuality education and contraceptive use. All girls and parents/guardians who responded to the relevant questions in the 4th follow up round were included in the analyses for this study.

### Measures

#### Occurrence of parent-child communication about sexual issues

The dependent variable for the current study was the occurrence of parent-child sexuality communication. Girls were asked “How many times have you talked about romantic relationships or sexual issues (including abstinence, sex, condoms and contraceptives) with your guardians?”, and the response options were “5 or more times” (1), “2–4 times” (2), “once” (3) “never”(4), or “I don’t know” (5). Because the majority of the respondents reported ‘never’ having communicated with their parents about sex (62%, see Additional file 2), a dichotomous variable was created ‘*Ever communicated with parents about sexuality issues*’ (1 = Yes, 0 = No); the “I don’t know” responses were excluded from the analyses. The distribution of responses to the below variables, according to communication frequency, can also be seen in Additional file 2.

#### Sociodemographic variables

The sociodemographic variables for girls included age and school enrolment. For parents, variables included age, sex and level of education. For the regression analyses, the parental level of education was grouped in to three levels, ‘Primary school and/or no formal education’; ‘Secondary level’; ‘Diploma and/or university’.

#### Psychosocial variables

A total of seven psychosocial characteristics from parents’ (three variables) and girls’ (four variables) interviews were examined. Variables related to the nature of the parent-child relationship, style of parents’ communication, parents’ attitudes and daughters’ *perceptions* of parental attitudes. Response formats for the questions varied between “Yes/No/I don’t know” and 5-point scales “strongly agree; agree; neither agree nor disagree; disagree; strongly disagree”. All five-point response options were reduced to ‘Yes’, ‘No’ and ‘Don’t know’.^5^

##### Parent-child connectedness

Connectedness refers to the extent to which a child perceives their parents to be caring, interested and responsive to them [45]. Thus, the variable *‘Girl’s Connectedness’* was based on girls’ level of agreement with the statement “When I speak to my parents/guardians about my problems or worries, I feel they really understand me”.

##### Parental comfort in communicating about sexual issues

Parents’ comfort in communicating about sexual issues was measured from parent’s self-reports as well as girls’ perceptions of their parents’ comfort. ‘*Parents’ Comfort’* indicated parents’ self-reported response to “Do you feel comfortable talking to your daughter about romantic relationships and sexual issues?”. ‘*Girl’s perceived parental comfort’* measured girls’ responses to the statement “My parents/guardians are comfortable with speaking to me about romantic relationships and sexual issues”.

##### Fear-based communication

The variable ‘*Girl-reported fear-based communication’* assessed whether the girls felt that parents used fear-laden messages in their communication about sexual issues. This was based on the girls’ level of agreement with “When my parents speak to me about romantic relationships and sexual issues, I feel that they try to scare me”.

##### Parental attitudes

Three variables refer to parental attitudes towards adolescent sexual and reproductive health – two were self-reported in parent interviews and one from girl’s interviews. ‘*Parent’s daughter ready for Sexual and Reproductive Health (SRH) education’* was based on the question, “Do you think your daughter is ready to learn about sexual and reproductive health issues?”. ‘*Parent’s perception of contraceptive harm’* was based on “Do you think it is harmful for a girl who has reached puberty and who is sexually active but not married to use contraceptives, for example injections/pills?”. The third variable assessed girls’ *perception* of parents’ attitudes; ‘*Girl perceives parent as objecting to contraception education’* was based on responses to “My parents or guardians think it is harmful for me to learn about condoms and other contraceptives.”.

### Data analysis

Pooled data using all the three study arms in the RISE 4th follow-up interview was used for the cross-sectional analyses in this study; STATA software version 15.1 (Stata Corporation, College Station, TX, USA) was used. Robust standard errors were included in all analyses to account for the cluster design. Univariate and multivariable logistic regressions were used to test the associations between the occurrence of parent-child communication about sexual issues and the independent variables described above. To control for sociodemographic variables, three separate models were run; Model 1 included sociodemographic variables only, Model 2 included psychosocial variables only and Model 3 included all the variables. The crude and adjusted odds ratios (AOR) with 95% confidence intervals (CI) are reported for all the associations. For comparative purposes, the three regression models were also run on each of the three study arms- the two intervention groups and the control. The results for the stratified sample are discussed in brief below and the complete table of results can be found in an additional file (see Additional file 3). To test for the level of agreement between parents’ self-reported level of comfort and girls’ perception of parental comfort, inter-rater reliability analysis using Cohen’s Kappa was carried out.

## Results

### Descriptive results

As shown in Table 1, a total of 4343 girls ranging from 11 to 27 years old were interviewed (Median age 15). Of the girls interviewed, 88% were currently enrolled in school and 35% had ever communicated with their parents about issues relating to sexual and reproductive health. As shown in Table 2, most girls reported connectedness with their parents (76%), nearly half reported that their parents used fear-based communication. Half of the girls perceived their parents to be against contraception education. A slight minority of the girls perceived their parents as comfortable when communicating about sexual issues.

**Table 1:** Sample characteristics

Girls’ characteristics	
*N*^*a*^	4343
*In School – N (%)*	
Yes	3812 (88%)
No	531 (12%)
*Median Age (IQR)*	15 (IQR:15–16)
*Married*	
Yes	135 (3%)
No	4205 (97%)
*Ever given birth*	
Yes	423 (10%)
No	3919 (90%)
*Ever communicated with parents about sexuality* (‘don’t know’ respondents excluded) *– N (%)*	
Yes	1521 (35%)
No	2713 (65%)
**Parent characteristics**	
*N*	3878
*Sex – N (%)*	
Male	870 (23%)
Female	2990 (77%)
*Parent’s level of education– N (%)*	
No formal education	344 (9%)
Primary	1813 (47%)
Junior secondary	893 (23%)
Senior secondary	581 (15%)
Certificate/diploma	175 (4%)
University	71 (2%)
*Relationship to child*^*b*^*– N (%)*	
Parent	2778 (72%)
Guardian	548 (14%)
Other	552 (14%)

^a^These numbers indicated do not include ‘missing’ and ‘non response’ cases

^b^For the analysis, reference to parents and their responses include all three of these relationship types

**Table 2:** Response to psychosocial variables

Psychosocial variables	Yes	No	Don’t know
*Girls’ measures*			
Girl’s connectedness	3308 (76%)	918 (21%)	115 (3%)
Girl-reported fear-based communication	2095 (48%)	2104 (49%)	142 (3%)
Girl’s perceived parental comfort	1958 (45%)	2306 (53%)	76 (2%)
Girl perceives parent as objecting to contraception education	2160 (50%)	2059 (47%)	122 (3%)
*Parents’ measures*			
Parent’s comfort	2866 (76%)	906 (24%)	15 (0.4%)
Parent’s daughter ready for SRH education	2153 (57%)	1498 (39%)	138 (4%)
Parent’s perception of contraceptive harm	2235 (59%)	1446 (38%)	101 (3%)

Of the 3878 parents/guardians interviewed, the majority were female and most of the respondents had education levels at either primary school or below. Most parents reported being comfortable when communicating about sex-related issues with their children and more than half (59%) believed that contraceptives are harmful for girls. A slight majority of parents believed that their daughters were ready to learn about sexual and reproductive health (SRH) issues.

When including all parental respondents, inter-rater reliability analysis showed a weak level of agreement between parental self-reported comfort and daughter’s perception of parental comfort (K = 0.0407, *p* = 0.0016) [53]. In order to ascertain whether the sex of the parent or the relationship to the child impacted the inter-rater reliability, separate analyses were carried out for the different groups of respondents– ‘parents’, ‘guardians’ and ‘other’ – as well as males and females. Amongst the three groups, although weak, only parents’ self-reported comfort had a statistically significant level of agreement with daughters’ perception of comfort (k = 0.450, *p* = 0.0031). Similarly, only female guardians’ self-reported comfort had a weak but significant level of agreement with daughters’ perception of their comfort (k = 0.0412. *p* = 0.0033).

### Cross-sectional associations between variables

The odds ratios and 95% confidence interval (CI) for the univariate and multivariable logistic regression analyses are shown in Table 3 and the results are described below. Model 1 included only the sociodemographic variables. Of these, girls’ school attendance was the only variable which was associated with the occurrence of parent-child communication about sexual issues (AOR 0.56, 95% CI 0.44–0.71); out of school girls were more likely to communicate with their parents about sexual issues (48.3%) than those who were in school (34.2%). Girls’ age was associated with communicating about sexual issues in the univariate regression (OR 1.08, 95% CI 1.03–1.13), but not when it was combined with the other variables. With regards to parents’ level of education, the results are indicative of an association; in comparison to those with primary or no education, parents with higher level of education (Diploma or university level) tended to have lower odds of communicating with their children about sexual issues than those with secondary school education level.

**Table 3:** Associations between occurrence of parent-child communication about sexual issues and sociodemographic and psychosocial variables

	Ever communicated with parents about sexuality issues (%)	Crude Odds Ratio (95% CI)	Model 1* Adjusted Odds Ratio (95% CI) *N* = 3545	Model 2** Adjusted Odds Ratio (95% CI) *N* = 3473	Model 3*** Adjusted Odds Ratio (95% CI) *N* = 3452
**Sociodemographic variables**					
*Girl age*		1.08 (1.03–1.13)	1.04 (0.98–1.10)		1.03 (0.97–1.09)
*Girl in school*					
Yes	34.2%	0.55 (0.45–0.67)	0.56 (0.44–0.71)		0.54 (0.42–0.69)
No	48.3%	Ref.	Ref.		Ref.
*Sex of parent*					
Female	36.7%	0.99 (0.84–1.17)	1.01 (0.86–1.20)		1.03 (0.87–1.22)
Male	36.8%	Ref.	Ref.		Ref.
*Parent’s education level*					
Primary or none	36.4%	Ref.	Ref.		Ref.
Secondary level	37.9%	1.06 (0.92–1.22)	1.10 (0.96–1.28)		1.09 (0.93–1.26)
Diploma or University	30.2%	0.75 (0.55–1.03)	0.82 (0.60–1.14)		0.71 (0.51–1.00)
**Psychosocial variables**					
***Girls’ measures***					
*Girl’s connectedness*					
Yes	36.7%	1.25 (1.04–1.49)		1.24 (1.01–1.52)	1.26 (1.03–1.55)
No	31.7%	Ref.		Ref.	Ref.
Don’t Know	47.7%	1.96 (1.20–3.20)		2.06 (1.23–3.47)	2.0 (1.21–3.41)
*Girl-reported fear-based communication*					
Yes	32.6%	0.73 (0.64–0.83)		0.76 (0.65–0.89)	0.72 (0.61–0.85)
No	39.7%	Ref.		Ref.	Ref.
Don’t Know	27.1%	0.56 (0.37–0.85)		0.70 (0.43–1.13)	0.63 (0.38–1.04)
*Girl’s perceived parental comfort*					
Yes	50.74%	3.31 (2.80–3.92)		2.94 (2.45–3.54)	2.98 (2.47–3.60)
No	23.7%	Ref.		Ref.	Ref.
Don’t Know	28.6%	1.28 (0.68–2.41)		0.98 (0.53–1.80)	1.02 (0.55–1.89)
*Girl perceives parent as objecting to contraception education*					
Yes	31.6%	0.68 (0.58–0.80)		0.76 (0.63–0.91)	0.77 (0.64–0.93)
No	40.3%	Ref.		Ref.	Ref.
Don’t Know	38.8%	0.94 (0.61–1.44)		0.72 (0.41–1.25)	0.73 (0.42–1.27)
***Parents’ measures***					
*Parent’s comfort*					
Yes	36.5%	0.97 (0.81–1.17)		0.86 (0.70–1.05)	0.86 (0.70–1.05)
No	37%	Ref.		Ref.	Ref.
Don’t Know	35.7%	0.94 (0.30–2.90)		0.80 (0.23–2.78)	0.92 (0.27–3.16)
*Parent’s daughter ready for SRH education*					
Yes	37.8%	1.12 (0.96–1.31)		1.09 (0.92–1.30)	1.07 (0.90–1.28)
No	35%	Ref.		Ref.	Ref.
Don’t Know	35.8%	1.03 (0.70–1.52)		1.01 (0.66–1.54)	0.95 (0.62–1.45)
*Parent’s perception of contraceptive harm*					
Yes	36.3%	0.96 (0.83–1.12)		0.98 (0.84–1.14)	0.98 (0.84–1.15)
No	37.1%	Ref.		Ref.	Ref.
Don’t Know	37.9%	1.03 (0.64–1.67)		0.92 (0.56–1.53)	0.91 (0.55–1.50)
*Model fit estimates*					
AIC			4636.955	4308.206	4255.484
BIC			4673.995	4400.498	4378.418

*Model 1- Psychosocial variables; **Model 2- Sociodemographic variables; *** Model 3- Sociodemographic and psychosocial variables

Model 2 included only the psychosocial variables. The variables assessed from the girls’ interviews were the only ones that showed a significant association with occurrence of parent-child communication about sexual issues. The girls’ perception of their of parents’ comfort seemed to be an important determinant for parent-child communication about sexual issues; girls who perceived their parents to be comfortable in communicating about sexual issues were more likely to communicate with their parents about sex than those who did not had (AOR 2.94, 95% CI 2.45–3.54). Similarly, girls who felt connectedness with their parents were more likely to communicate with them about sexuality issues than those who did not (AOR 1.23, 95% CI 1.01–1.52). Those girls who were unsure about the level of connectedness with their parents (‘don’t know’), were also more likely to communicate with their parents than those who responded ‘no’ (AOR 2.06, 95% CI 1.23–3.47). Conversely, girls who indicated that their parents use fear-based communication about sexuality, were less likely to communicate with their parents about sexual issues than those who did not report fear-based communication (AOR 0.76, 95% CI 0.65–0.89). The girls who perceived their parents as objecting to contraceptive education were also less likely to communicate with them about sexual issues (AOR 0.76, 95% CI 0.63–0.91).

Model 3 combined all the independent variables, both sociodemographic and psychosocial. The pattern of associated variables remained unchanged; all variables which were associated with parent-child communication about sexual issues in Models 1 and 2, were also associated with the dependent variable when combined. The five variables associated with occurrence of parent-child communication were: ‘girls’ school attendance’, ‘girl’s connectedness’, ‘girl-reported fear-based communication’, ‘girl’s perceived parental comfort’ and ‘girl perceives parent as objecting to contraception education’. According to the Akaike information criterion (AIC) Bayesian information criterion (BIC) estimates it seems that Model 3 provides the best predictive fit.

Additional analyses were carried out to ensure that the associations seen in the pooled sample were not masking important differences between the study arms. Regression Model 3 (which included all sociodemographic and psychosocial variables) was run on the data from each of the three study arms separately and compared with the results from the pooled sample (see Additional file 3). The associations between parent-child communication about sexual issues and the sociodemographic and psychosocial variables were of largely similar strength and direction across the study arms and the pooled sample. However, in both the control arm and the combined intervention arm, there seemed to be a lower likelihood of diploma/university educated parents communicating with their children about sex than secondary school educated parents (Control Arm: Diploma/university: AOR 0.26, 95% CI 0.09–0.73; Secondary school: AOR 1.00, 95% CI 0.73–1.36; Combined Intervention Arm: Diploma/university: AOR 0.61, 95% CI 0.37–1.00; Secondary school: AOR 1.04, 95% CI 0.85–1.27. Similarly, the pooled data showed no association between communication occurrence and parents’ (self-reported) comfort, but in the control arm, communication about sexual issues tended to be more likely if parents felt comfortable communicating with their children about sex, than if they were not (AOR 1.45, 95% CI 0.92–2.27). Conversely, in the combined intervention arm, parents’ comfort was negatively associated with communication about sexual issues (AOR 0.69, 95% CI 0.52–0.92).

To further examine the association between school going and communication about sexual issues, a post-hoc multivariate logistic regression was carried out between school-going status (dependent variable) and age and sexual debut (yes/no). The results suggest that both age and sexual debut was associated with school enrolment. That is, in-school girls were younger (AOR 0.64, 95% CI 0.58–0.72) and less likely to have had their sexual debut than those out of school (AOR 0.045, 95% CI 0.03–0.06).

## Discussion

Within this sample, less than half of girls had communicated with their parents about romantic and/or sexual issues and those out of school were more likely to have done so. Neither the girls’ age, sex of the parent, or parents’ education level were (significantly) associated with whether parent-child communication about sexual issues took place. However, girls who felt connected with their parents were more likely to communicate with them about sexual matters than those who did not. Likewise, girls who perceived their parents as comfortable in communicating about sexual issues, were also more likely to have communicated with them about such topics. However, girls who perceived their parents to be opposed to them learning about contraception and those whose parents used fear-based communication around sex, were less likely to have communicated with them about sex than those who did not. None of the parents’ self-reported attitudes were associated with the occurrence of parent-child communication about sexual issues in any of the models. Although there was a weak correlation between parental self-reported comfort and daughters’ perception of parental comfort, this was only significant when the respondent was indicated as the parent, as opposed to a guardian or other. Agreement between parents’ self-reported and daughters’ perceived parental comfort was only seen when the parent/guardian was a female. Although cross sectional data do not allow for causal inference, the results may suggest that the nature of the parent-child relationship and the way in which parents communicate with and convey their attitudes towards sexual issues, can influence the likelihood of them communicating with their children about sex-related topics.

The proportion of adolescent girls reporting parent-child communication about sexual issues in this sample (35%) is comparative to findings from other countries in sub-Saharan Africa [17]. Although low, these figures can be optimistically interpreted because they suggest that despite social and cultural taboos around parent-child communication about sex in SSA [18], some parents *are* communicating about sexual issues with their children. This also means that there is scope for increasing both the amount and quality of parent-child communication about sexual issues as a means of addressing risky sexual behaviour amongst adolescents in the region.

The findings confirm the importance of considering the quality of the parent-child relationship when considering involving parents in addressing adolescent sexual risk behaviour. A child who feels that her parents are interested in and understand her problems (connectedness) is more likely to seek advice and guidance about important sensitive issues, such as sex [54]. Indeed, this has been demonstrated in a South Africa study, which showed that when the parent-child relationship was strengthened, it also increased the frequency of parent-child communication about HIV/AIDS and sexuality issues [55]. It seems therefore that parent-based interventions would benefit from focusing on increasing connectedness between parents and their children.

The results of this study also suggest that efforts to increase parent-child sexuality communication may benefit from helping parents to reduce fear-based messages when talking to their children about sexual issues. Indeed, interventions have demonstrated that parents can be trained to change their communication styles from passive-aggressive communication to providing assertive and direct messages about sexual issues [37, 49, 56]. This may be particularly relevant in sub-Saharan Africa where qualitative studies in several countries have found that that fear-based messages and a fear of physical punishment prevents adolescents from speaking to their parents about sexual issues [20, 49, 50, 57]. This study gives strength to suggestions that efforts to reduce adolescent sexual risk in SSA countries such as Zambia, may not only benefit from training parents to communicate with their children about sexuality issues [24], but that specific focus should be placed on the formulation of neutral and open messages about sexual issues.

The importance of parents’ comfort in communicating about sexual issues was confirmed in this study. However, it was interesting to note that only the girls’ *perception* of their parents’ comfort (and not parents’ self-reported comfort) was associated with parent-child communication. Although most parents reported being comfortable in communicating about sexual issues, they were not more likely to communicate with their daughters about sex than those who were not. One explanation could be socially desirable responding; parents may be over-stating their comfort level because they do not want to appear as if they are intimidated by their own children. However, another explanation may be that the child’s *perception* of the parent’s comfort is in fact more important than whether the parent him/herself feels comfortable. This interpretation can also be applied to the finding that none of the parents’ self-reported attitudes were associated with the occurrence of communication about sexual issues, but the girls’ *perception* of parents’ attitudes was. It may be that in contexts where open and direct communication between parents and children is less common (as in parent-child communication about sexual issues), adolescents rely more on their perceptions of their parents’ attitudes than on what their parents communicate to them directly.

Few studies have quantitatively measured the association between the child’s *perception* of their parents’ attitudes and the likelihood of them communicating with their parents about sex. Although several interventions, including the RISE study, address parents’ attitudes towards adolescent sexual and reproductive health, it may be that children’s perceptions of their parents’ attitudes is equally important with regards to communicating about sexual issues. Indeed, the data in this study suggest that this is the case; adolescent girls who perceive their parents as holding negative attitudes towards education about contraceptives, were less likely to communicate with them about sexual issues. The objection to and/or fear of modern contraceptives is a commonly observed narrative in the SSA region [58] where topics such as condoms and contraception are often omitted from discussions in preference to topics such as puberty, abstinence and HIV [15, 20, 22, 27, 38, 48, 59, 60]. This is often attributed to cultural beliefs and practices, but in many cases may be the result of lack of knowledge about contraception amongst parents [20, 58]. Considering that accurate information on contraception is essential to promoting safe sexual behaviour, parent-child communication interventions may benefit from challenging parents’ negative attitudes towards contraception and encourage parents to display more open attitudes when communicating with their adolescents about sex.

The finding that out-of-school girls were more likely to communicate with their parents contradicts previous studies from surrounding countries. For example in a study in Tanzania, girls in school reported more HIV communication with parents than those out of school [61]. However a number of studies in Kenya, Tanzania and Nigeria also suggest that misconceptions amongst parents prevent them from talking to their children about sex whilst they are in school ([20–22]). These studies found that parents tend to believe that girls who are in school do not have sex and thus do not need information about it [20]. Parents were also more likely to speak to their children about sex when they are older, reach puberty/menses and/or when parents suspect they have romantic relationships and exhibit signs of engaging in sexual behaviour [35, 37, 54]. However, as suggested by the formative studies conducted for the RISE project [62], it is also possible that out of school girls in rural Zambia perceive themselves as adults who are ready for marriage and therefore are less hesitant to speak to their parents about sexual issues. This aligns with the results of the post-hoc analysis which suggested that out-of-school girls, were more likely to be older and more likely to have engaged in sex than those in school.

A number of studies in countries in SSA suggest that parents with higher educational level are more likely to communicate with their children about sexual issues [27, 60, 61]. The results from this sample concur with this; parents with a high level of educational attainment (diploma or university) were less likely to communicate with their children about sex than those with secondary-school level education. This is an interesting finding and may suggest that parents who are highly educated have higher ambitions for their children and are even more motivated to ensure that their daughters are not sexually active and remain in school. In a cultural setting where communicating about sex is thought to encourage sexual activity, is possible that highly educated parents with higher ambitions for their children, are even more hesitant to communicate with their children about sexual issues than those with lower levels of education.

### Limitations

The cross-sectional nature of the analyses in this study does not allow for causal inferences between the independent and dependent variables. However, the results do provide us with an indication of the characteristics of communication that are worth investigating further. This will be possible using data from future rounds of follow-up interviews from the RISE project, as they become available. These data will also allow us to examine how parent-child communication about sexual issues is related to sexual debut and contraceptive use amongst girls. These analyses will be possible in 2020, when the RISE project comes to an end.

Grouping the response options from five-points to three options (Yes/No/I don’t know) reduced the level of detail of the data. In addition, measuring complex constructs such as comfort and parent-child connectedness using only one scale item, is likely to have implications for the precision and validity of the constructs measured. By using tested and validated scales (using multiple questions) to measure the psychosocial constructs, a more nuanced understanding of the construct would have been possible. Although the number of additional interview items that could be included in the RISE follow-up was restricted, future studies have the possibility to study these characteristics more in-depth.

All the interview questions were designed with local experts and piloted before implementation. However, the risk of socially desirable responding is ever-present in self-reported data and especially so when studying sensitive issues. In addition, the data for this study was collected using face-to-face interviews which may have increased the chances of socially desirable responding. In consecutive follow-up rounds of the RISE study, numerous steps have been taken to reduce socially desirable responding, including the use of Audio Computer Assisted Survey Instruments (ACASI).

Finally, the generalizability of the results of this study must be approached with caution; firstly, the all-female sample in this study must be considered. Given that parent-child communication about sexual issues has been found to be consistently lower amongst males, further studies are needed to establish whether the patterns of associations found in this study vary between males and females in other contexts. If considerable and consistent differences are found between males and females, this may inform the different ways in which parents can increase communication about sexual issues with their sons and daughters. Secondly, the data for this research comes from an intervention study in which more than 80% of the sample were exposed to conditions aimed at reducing adolescent sexual risk behaviour, meaning that the findings may not be representative of parent-child communication about sexual issues in other populations. In particular, the girls and parents in the combined intervention arm were directly exposed to activities aimed at sensitizing them and increasing their knowledge on aspects of safe sexual behaviour for adolescents. These activities are likely to have impacted on their knowledge and attitudes around adolescent sexual behaviours and thus their levels of comfort, the likelihood of them discussing sexual issues and the ways in which they do so.

## Conclusion

This study revealed specific sociodemographic and psychosocial factors which may influence whether parents speak to their children about sexual issues. Specifically, the findings indicate that the likelihood of parent-child communication about sexual issues is greater when children feel listened to and understood by their parents (connectedness) and when they perceive their parents to be comfortable in communicating about sexual issues. Conversely, the likelihood of parent-child communication about sexual issues is reduced when parents use fear-based communication and when children perceive their parents as holding negative attitudes towards contraception. Given the potential that parent-child communication about sexual issues may have in reducing adolescent sexual risk, the findings suggest the need to address those aspects that prevent parent-child communication about sexual issues in Zambia and other countries in sub-Saharan Africa [49]. Although in-depth analysis is required to better understand the local attitudes and discourses around adolescent sexual and reproductive health in Zambia; the results from this study suggest that interventions to increase the likelihood of parent-child communication about sexual issues may benefit from encouraging parents to convey non-judgemental attitudes and use open communication styles which do not use fear to discourage adolescents from having sex. Interventions may also benefit from considering the ways in which parents are perceived by their children when they communicate with them about sex.

## Supplementary information

**Additional file 1.** Questionnaires used for Girls’ and Parents’ 4th round follow-up interviews. Questionnaires developed for the interviews with participants and parents for the 4th round of follow up in RISE (on which the data for this paper was collected). The questionnaire was developed in collaboration with the Zambian and Norwegian research teams and is not published in any other location).

**Additional file 2 **Additional data on distribution of responses for *occurrence of parent-child communication about sexual issues* and psychosocial variables used in the study. Table 1 presents the distribution of responses to *‘Occurrence of parent-child communication about sexual issues’* broken down by the five original response categories. This shows the distribution of responses before the variable was converted to a dichotomous variable. Table 2 shows the response patterns for the psychosocial variables according to the five original response options for the frequency of parent-child sexuality communication (“How many times have you talked about romantic relationships or sexual issues.”).

**Additional file 3.** Comparison of Model 3 results (sociodemographic and psychosocial variables) between the three groups in the RISE study (Control, Economic intervention, Combined intervention) and the pooled data. The table shows the results of the multivariate regression for Model 3 – which included both the psychosocial and sociodemographic variables – when the data was disaggregated according to the three groups in the RISE study: the control group, the economic intervention and the combined intervention group. OR and AORs are included for each of the variables in each of the groups. For comparison, the regression results for the pooled data (as presented in the paper) are also included/repeated in the table.

## Data Availability

The datasets used and/or analysed during the current study are available from the corresponding author on reasonable request.

## Abbreviations

AOR: Adjusted Odds Ratio
ASRH: Adolescent Sexual and Reproductive Health
ACASI: Audio Computer Assisted Survey Instruments
CISMAC: Centre for Intervention Science in Maternal and Child Health
CI: Confidence Interval
GLOBVAC: Global Health and Vaccination Programme
HIV: Human Immunodeficiency Virus
RISE: Research Initiative to Support the Empowerment of Girls
SRH: Sexual and Reproductive Health
SSA: Sub-Saharan Africa
UNZA: University of Zambia

## Footnotes

1 This text assumes UNICEF’s definition of an adolescent; an individual between the age of 10 and 19 years of age

2 The term parent and guardian are used interchangeably in the text.

3 At recruitment 6 girls were married and 20 had given birth.

4 Parents had given their informed assent to be interviewed when giving consent for their daughters’ enrolment at the start of the study.

5 “Strongly agree” and “agree” were coded as ‘Yes’; “Neither agree nor disagree” was coded as ‘Don’t Know’ and “Disagree” or “Strongly disagree” were coded as ‘No’
