# Supplementary material for: Parent-child communication about sexual issues in Zambia: a cross sectional study of adolescent girls and their parents
Source: BMC Public Health. 2020 Jul 16;20:1120. doi: 10.1186/s12889-020-09218-y (PMC7364553; doi:10.1186/s12889-020-09218-y)
Supplement: Supplementary file 1 — Additional file 1. Questionnaires used for Girls’ and Parents’ 4th round follow-up interviews. Questionnaires developed for the interviews with participants and parents for the 4th round of follow up in RISE (on which the data for this paper was collected). The questionnaire was developed in collaboration with the Zambian and Norwegian research teams and is not published in any other location). [file 12889_2020_9218_MOESM1_ESM.docx]

## Additional File 1: Questionnaires used for Girls’ and Parents’ 4^th^ round follow-up interviews (developed in collaboration by Zambian and Norwegian research team)

### **4^th^ Round follow up Interview for Girls**

Research Assistant Name:­­­­­­­­­­­­­­ ____________________________

Date of Interview: __________________________________

**Visit one**

Select name of girl from case list

Girl found?

- Yes
- No, not found at school or at home.

Appointment made?

- Yes
- No

If no, why not? ____________________

__:__ hours on __/__/_____ [date]

Place to meet girl ________________

When the girl has been found, verify her identity with teachers or family members. Mention that if the right girl is not around, you can come back on another day.

Greetings. I am from the RISE project. My name is  **xx**.

Are you [name of girl]?

- - Yes
  - No -> Find out whether the girl you want to speak to is around. If not, end interview and attempt to make appointment

1. I am one of the research assistants from the RISE project. The project needs to keep in touch with the participants regularly to find out how and what they are doing. I am going to ask you a few questions which will take about 10 minutes. Is this a convenient time to talk?
   - Yes -> iii)
   - No ->ii)
2. When will be a good time to see you for the interview?

__:__ hours on __/__/_____ [date].

Tell her you will be coming back. -> End interview

1. I would like to emphasise that whatever information you share with me, it will not be shared with others together with your name. That means that the information you provide will be put together with information from many other girls coming from all the other schools in the project, and your name will not appear in any reports. Your personal information will not be shared with anyone else. You will continue receiving support from the project no matter how you respond to the questions, or if you have dropped out of school, or have got married or have fallen pregnant.

**Before we start I need to ask you some questions to make sure that I am talking to the right person**

The correct response will appear in the form.

A)

1. What is your date of birth? (day-month-year)
2. Correct response?
   - Yes
   - No
3. How confident are you about your date of birth
4. Does the school enrolment register give ecact date of birth, or just year of birth?
5. Which year of birth is in the school register?
6. Were you interviewed by someone from the RISE-project between September and December 2017?

**START OF FOLLOW-UP INTERVIEW**

1. Do you live in the same place as you did the last time you were interviewed or have you moved to a different place?

- - Lives in the same place
  - Has moved

If she lives in the same place, skip q2 -5

2. What is the name of the new village where you live? _____________________

3. What is the name of the new district where you live?

_______________________

4. Please mention a special landmark or popular place close to your new home that can guide someone who does not know where you live?

__________________________

**Personal Information**

1. Are you currently going to school?
   - Yes
   - No

If no, skip questions 6 and 7.

1. A Which school are you enrolled in? _______________

6B Which grade are you enrolled in? (range 7-9)

If enrolled in grade 8 or 9 and in group II and III, ask 6C:

6C Did the RISE project pay school fees for you this year?

1. How many days did you go to school last week?

If no to q 5 or zero to q7, ask q 8 and 9

1. When did you last attend school? ______________________(Response format: mm/yyy)
2. Which grade were you enrolled in just before you stopped going to school? ______

If in group II and III, ask 10-14:

**I am now going to ask some questions about your experiences with the RISE project**

1. In the last 6 months, did you receive the monthly cash transfer from the project?
   - Yes, every month
   - Yes, some months
   - No
   - Don’t know
2. When did you last receive cash from the RISE project? ___ (enter month and year)

If no to q10, skip question 12-14

1. The last time you received the monthly transfer, how much money did you receive? ___________________
2. Did you give some of the monthly transfer to your guardians/parents or did you keep all of it to yourself?

- Gave to parents/guardians
- Gave to others. Specify______________
- Kept all of it

1. A How much did you give to your guardians/parents

14. B Have you spent the monthly transfers on any of the following items?

Read all options:

- 1. Pencil
  2. Pen
  3. Rubber
  4. Books
  5. Snacks
  6. Lotion
  7. Make-up
  8. Other, specify__

To all

15a Have you ever learned anything about sexual and reproductive issues at school?

- 1. Yes
  2. No -> skip 15b

15b Did you learn about sexual and reproductive issues at school last year?

- 1. Yes
  2. No

16a Have you participated in any RISE meetings over the last 6 months?

- 1. Yes
  2. No -> skip 16b

16b Which meetings have you attended? *(several can be ticked)*

- 1. Youth club meetings
  2. Community meetings
  3. Bicycle lottery
  4. Other, specify________________

If has not attended youth club meetings, skip q17.

1. How many youth club meetings have you attended in the last school term?

__________ meetings

To all:

We would like to learn from you how you experience being part of the RISE project.

1. Have you experienced any good things as a result of your participation in this RISE project?
   - Yes
   - No ->skip q19
2. What have you experienced?
3. Have you experienced any problems as a result of your participation in the RISE project?
   - Yes
   - No -> skip q21
4. What have you experienced?

_____________________

**I am now going to ask you some questions regarding sexual and reproductive health issues such as abstinence, HIV, pregnancy and contraceptives. I will start out by asking you about who you discuss sexual and reproductive issues with**

1. Who do you prefer to talk with about romantic relationships or sex? (Tick those the girl mentions spontaneously. More than one option may be selected.)

- Mother
- Father
- Older sister
- Younger sister
- Brother
- Aunt
- Grand mother
- Cousin
- Friends
- Boyfriend
- Teachers
- Formal health workers
- Community health workers
- Other, specify
- No one

1. Who do you prefer to talk with about condoms and other contraceptives? (Tick those the girl mentions spontaneously. More than one option may be selected.)

- Mother
- Father
- Older sister
- Younger sister
- Brother
- Aunt
- Grand mother
- Cousin
- Friends
- Boyfriend
- Teachers
- Formal health workers
- Community health workers
- Other, specify
- No one

1. How many times have you talked about romantic relationships or sexual issues (including abstinence, sex, condoms and contraceptives) with your boyfriend or another sexual partner?

- 5 or more times
- 2-4 times
- Once
- Never
- Does not have a boyfriend

1. How many times have you talked about romantic relationships or sexual issues (including abstinence, sex, condoms and contraceptives) with your guardians?

- 5 or more times
- 2-4 times
- Once
- Never

I am now going to read to you a series of statements about communication about romantic relationships and sexual issues. The statements are phrased as “I” and “my parents” but they are about you and your parents and how you communicate about romantic relationships and sexual issues.I am interested in your opinion, and no answers are more correct than others. For each statement, please tell me if you strongly agree, agree, neither agree nor disagree, disagree or strongly disagree.

1. My parents/guardians are comfortable with speaking to me about romantic relationships and sexual issues

- Strongly agree/agree very much
- Agree
- Neither agree nor disagree
- Disagree
- Strongly disagree/disagree very much

1. When my parents speak to me about romantic relationships and sexual issues, I feel that they try to scare me.

- Strongly agree/agree very much
- Agree
- Neither agree nor disagree
- Disagree
- Strongly disagree/disagree very much

1. When I speak to my parents/guardians about my problems or worries, I feel they really understand me.

- Strongly agree/agree very much
- Agree
- Neither agree nor disagree
- Disagree
- Strongly disagree/disagree very much

1. My parents or guardians think it is harmful for me to learn about sexual abstinence.

- Strongly agree/agree very much
- Agree
- Neither agree nor disagree
- Disagree
- Strongly disagree/disagree very much

1. My parents or guardians think it is harmful for me to learn about condoms and other contraceptives.

- Strongly agree/agree very much
- Agree
- Neither agree nor disagree
- Disagree
- Strongly disagree/disagree very much

1. If I became pregnant now, my parents would expect me to get married.

- Strongly agree/agree very much
- Agree
- Neither agree nor disagree
- Disagree
- Strongly disagree/disagree very much

I am now going to ask you some questions where I will read out statements about things adolescent girls typically do. I would like you to listen to them and then tell me HOW MANY apply to you. Do not tell me which ones apply, just tell me how many. In order to make sure you understand what to do, I first give you an example of statements:

1. I listen to music every day
2. I read books every day
3. I like to cook
4. I help my siblings with their homework

After reading the statements, I want you to tell me how MANY of these are correct for you? None, one, two, (three ) or all. Is this clear?

1. I will now mention three/four things that adolescent girls typically do. Please listen to them and then tell me HOW MANY you have experienced. Do not tell me which ones, just tell me how many. Here are the three/four things:
2. I have visited the health clinic in the past year
3. I have been admitted to a hospital/clinic
4. I often have head ache
5. (Fourth item) I have been pregnant at least once

Now, how MANY of these have you experienced? None, one, two, (three ) or all?

1. Now I am going to read you another list. Please listen to all of the things and then tell me HOW MANY you have done. Again, not which ones, just how many.
2. I have had malaria in the past year
3. I have received injectable drugs
4. I have used herbal treatment for stomach cramps
5. (Fourth item) I have been pregnant at least once

Now, how MANY of these have you experienced? None, one, two, (three ) or all?

___________

**I now have some questions about childbearing**

34.Have you ever given birth?

- - Yes
  - No -> skip 35-47

35.When did you last give birth? ___(Date or month and year )

Skip this section if last gave birth before August 2017.

**Information about the occurrence of birth complications among girls who have recently given birth (within the past 6 months?)**

36.Was the baby born more than one month before the due date?

- Yes
- No
- Don't know

If answers yes, ask q37.

37.Can you estimate how many months the pregnancy was when you gave birth, based on your last menstrual period?---------------months

38 Did you give birth to one baby or twins?

- One (singleton)
- Twins

38.A Do you have an under-five card for the child?

- Yes
- No

38B How much did the baby weigh when he/she was born? *Record the weight in grams* ___

*Please enter the weight in grams. If the girl does not know, enter 89*

38Bii How much did the baby weigh when he/she was born? *Record the weight in grams*___

If yes to q38A, and the girl is interviewed at home, ask 38C:

38C Ask to see the under-five card and record the weight

Q38cii If the girl is interviewed at home, ask to see the under-five card and record the weight for baby 2

If she does not know the exact weight, ask q39, otherwise skip it:

39. Did the baby weigh less than 2500 g when he/she was born?

- Yes
- No
- Don't know

40. Is the baby alive?

- - Yes
  - No

41. Did you receive treatment for high blood pressure while you were pregnant?

- Yes
- No

If no, skip q42

42. Were you diagnosed with hypertension before you got pregnant?

- Yes
- No

43. Did you have any other complications?

- Yes
- No

44. Mention any other birth complication for which you received treatment? What happened?

- -------------------------------
- ........................................
- ........................................
- ........................................

45.Has the baby been admitted to a clinic or hospital over the last 6 months?

- Yes
- No

46. How many times has the baby been admitted to a clinic or hospital in the last 6 months?--------

47.What was the cause of the last admission?

- - - malaria,
    - diarrhea,
    - anemia,
    - Chest infection,
    - Other:--------------

I now have some questions about marriage and pregnancy

48. Are you married or living with a boyfriend?

- Yes
- No-> skip q76

49.When did you get married or start living with your boyfriend? Month _____ Year ______

Skip 50 if answers “yes” to question 34:

50. At what age do you think will be the right time for you to have your first child?

If yes to q34, skip 51

51. Have you ever been pregnant?

- Yes
- No

If no, skip q52

52. Are you currently pregnant?

- Yes
- No

If yes to 51 or 52, skip 53

53. Have you ever had sexual intercourse?

- - Yes
  - No

1. Which of the following contraceptives have you used to prevent pregnancy?
   1. Male condom
   2. Female condom
   3. Hormonal pill
   4. Intrauterine device
   5. Injectables
   6. Implants
   7. None
   8. Other, specify

**ACASI-part**

**Information about health care utilization**

1. Have you been admitted to a clinic or hospital in the last 6 months?

- yes
- No

If no, skip 2

2.Were you admitted due to pregnancy or birth related issues?

- - yes
  - No

3.Have you visited any outpatient clinic or pharmacy/drug shop or traditional birth attendant or healer in the last 6 months?

- yes
- No

If no, skip 4

4.What was your reason for visiting these places or people?

1. Did you visit these places or providers because of Contraception- yes/no
2. Did you visit these places or providers because of Sexually transmitted infection?- yes/no
3. Did you visit these places or providers because of pregnancy-related issues? yes/no
4. Did you visit these places or providers because of Child health issues?- yes/no
5. Did you visit these places or providers because of Malaria?- yes/no
6. Did you visit these places or providers because of other infections? - yes/no

5. Have you ever been pregnant?

- Yes
- No

If no, skip q6

6. Are you currently pregnant?

- Yes
- No

If no to q6, skip q 7

If yes to question 6, ask q 7-8

7. How many months do you think the pregnancy is now?

If yes to q 6, but no to q 7, ask q 8 and 9:

8. When did your last pregnancy end?

9. How did your last pregnancy end?

- Miscarriage/spontaneous abortion
- Induced abortion
- Stillbirth
- Live birth

Skip 10 if answers “yes” to question 6 or 7

10. Have you ever had sexual intercourse?

- 1. Yes
  2. No

Skip 11 if No to q10, but ask question to all others:

11. Have you ever used any contraception to prevent pregnancy?

- - Yes
  - No

Thanks a lot for responding to these questions! Please let the one who is interviewing you know that you are done with all the questions.

*END OF ACASI-part*

Thanks a lot for responding to these questions. Before we finish the interview, I would like to update your contact information.

To all:

If we don't find you next time we come to do interviews, who do you think can assist us in finding you?

Can you give us the name of your best friend?

55A) We need to make sure we have updated contact information. Which mobile phone number should we use if we need to call you in the future? _______________________________

B)Whose number is this?______________________

56A) Can you give us 2 other numbers that we can reach you on in case we cannot reach you on this number?

- - Yes-> B
  - Don’t know other phone numbers -> F

B) Provide first number ______________________________

C) Whose number is this?______________________

D) Provide second number_______________________________

E) Whose number is this?______________________

56. F If the girl is interviewed at home: After you and I have finished talking, may I ask your guardians for 2 alternative phone numbers we may try if we cannot get through on the numbers we have if we need to call you?

- Yes
- No

This is the end of the interview, thank you for taking time to answer my questions. We are very grateful. Your participation is extremely important for this project because it gives us an opportunity to understand how girls who receive the different support packages are faring.

For groups II and III:

Let me use the opportunity to provide some information about payment of school fees in 2018: We will not pay more in 2018 than we did in 2017. If you transfer, we will not be able to pay your school fees unless you inform us of where you have shifted to. Please call the Trial supervisor.

For all

Do you have any questions you may want to ask me before we finish the interview?

For group I:

As an appreciation of your collaboration, we would like to give you K50 as compensation for your time.

For groups II and III:

As an appreciation of your participation in this interview, we will give you a small compensation in term 3 of 2018. We will give you K40 if we have interviewed you for the three rounds since the end of 2017, i.e. the last round that was done in 2017 and the two that will be done in 2018. If we have interviewed you twice of the three times, you will get K20. If we have interviewed you once of the three times, you will get K10,

For all

Thank you very much again! We will contact you again in 4-6 months from now for another session of interview.

If yes to 55A, end interview here

If the girl is interviewed at home: Now I would like to ask your guardians for some additional phone numbers

Thank you very much for allowing us to speak to your daughter. I would like to find out whether you are able to give us 2 phone numbers that we can reach your daughter on in case we cannot find her when we visit next time in 4-6 months?

A) Provide first number______________________________

B) Whose number is this?______________________

C) Provide second number_______________________________

D) Whose number is this?______________________

Thank you so much! We will come to interview your daughter again in 4-6 months. Thanks a lot for your assistance!

If adverse events are reported, the research assistant should let the data manager know so that he can consider whether there is a need for the project to take action because of the adverse events reported.

Take a picture together with the girl and ask her to provide a thump print and a signature on your list to prove that you interviewed her.

Capture Location

### **4^th^ Round follow up interview for Parents**

Date of Interview

Time of starting interview

Place of interview

- School
- Home
- Other

Introduction:

Greetings. I am from the RISE project. My name is < user>.

Are you living in the same household as < girl_name>?

- Yes
- No

Ask whether she/he lives with a girl who is participating in the RISE trial, and if yes try to identify the name of the girl

A: Demographic information

Q1.Sex of guardian

- Male
- Female

Q2 What is your year of birth?

Enter 89 if does not know

Q4. Which of the following applies to you? *Read the options*

- Single
- Married
- Living together
- Divorced
- Widow/widower
- Other

Q4b. specify marital status

Q5.What is your relationship with < girl_name> ?

- Parent
- Guardian
- Sibling
- Grand parent
- Other

Q5b. specify relationship

Q6.Are you the head of the household?

- Yes
- No

Q7. What is the sex of the head of the household?

- Male
- Female

Q8.What is the highest level of education that the head of household ("you" if the respondent is the head of household) has attended?

- No formal education
- Primary
- Junior secondary
- Senior secondary
- Certificate/diploma
- University

Q10.What is the occupation of the head of household? ("you" if the respondent is the head of household)

- Self-employed agriculture
- Self-employed in (small or big) business/trade
- Employed
- Retired
- Daily wage laborer
- Not working
- Not earning any income
- Other

Q11. How many people usually live in your household now? Exclude visitors and children in boarding school

Q12. How many of these household members are children under the age of 5 years?

Check the response entered in Q12. It should not be higher than the response in Q11.

**B: FOOD EXPENDITURES**

Q13a. Who usually makes food purchases in the household? *More than option is possible*

- Respondent
- Someone else

Q13b. Specify
*Mention the RELATIONSHIP to the girl (RISE participant), NOT the name*

**Q14. I am going to mention a number of food items. In the last 7 days how much was the APPROXIMATE total cost of what was consumed of each of them? We want to know both about food which was purchased and about food that was produced at home. These foods may have been purchased or produced in the last 7 days or more. So when I refer to foods you have used in the last 7 days, please include foods that were carried over from last week in your answers. Do not include foods consumed in relation to funerals or weddings**.

14a. I am first going to ask you about different kinds of meat. I start with beef. In the last 7 days, did your household consume beef?

- Yes
- No

What do you estimate was the APPROXIMATE total cost of all the beef consumed in the last 7 days?
*Enter 999 if does not know*

Does this include the beef that was bought or produced earlier but was consumed in the last 7 days?

**If not, go back and adjust the amount**

b. In the last 7 days, did your household consume pork?

- Yes
- No

What do you estimate was the APPROXIMATE total cost of all the pork consumed in the last 7 days?

Does this include the pork that was bought or produced earlier but was consumed in the last 7 days? **If not, go back and adjust the amount**

c. In the last 7 days, did your household consume Chicken?

- Yes
- No

What do you estimate was the APPROXIMATE total cost of all the Chicken consumed in the last 7 days?
*Enter 999 if does not know*

Does this include the chicken that was bought or produced earlier but was consumed in the last 7 days? **If not, go back and adjust the amount**

d. In the last 7 days, did your household consume Fish?

- Yes
- No

What do you estimate was the APPROXIMATE total cost of all the Fish consumed in the last 7 days?
*Enter 999 if does not know*

Does this include the fish that was bought or fished earlier but was consumed in the last 7 days? **If not, go back and adjust the amount**

e. In the last 7 days, did your household consume Kapenta?

- Yes
- No

What do you estimate was the APPROXIMATE total cost of all the Kapenta consumed in the last 7 days?
*Enter 999 if does not know*

Does this include the kapenta that was bought or fished earlier but was consumed in the last 7 days? **If not, go back and adjust the amount**

f. In the last 7 days, did your household consume Rice?

- Yes
- No

What do you estimate was the APPROXIMATE total cost of all the Rice consumed in the last 7 days?
*Enter 999 if does not know*

Does this include the rice that was bought or produced earlier but was consumed in the last 7 days? **If not, go back and adjust the amount**

g. In the last 7 days, did your household consume Beans?

- Yes
- No

What do you estimate was the APPROXIMATE total cost of all the Beans consumed in the last 7 days?
*Enter 999 if does not know*

Does this include the beans that was bought or produced earlier but was consumed in the last 7 days? **If not, go back and adjust the amount**

h. In the last 7 days, did your household consume mealie meal?

- Yes
- No

What do you estimate was the APPROXIMATE total cost of all the mealie meal consumed in the last 7 days?
*Enter 999 if does not know*

Does this include the mealie meal that was bought or produced earlier but was consumed in the last 7 days? **If not, go back and adjust the amount**

k. In the last 7 days, did your household consume ground nuts?

- Yes
- No

What do you estimate was the APPROXIMATE total cost of all the ground nuts consumed in the last 7 days?
*Enter 999 if does not know*

Does this include the ground nuts that were bought or produced earlier but were consumed in the last 7 days? **If not, go back and adjust the amount**

l. Yesterday, did your household consume Sugar?

- Yes
- No

What do you estimate was the APPROXIMATE total cost of all the Sugar consumed yesterday?
*Enter 999 if does not know*

n. Yesterday, did your household consume Cooking oil?

- Yes
- No

What do you estimate was the APPROXIMATE total cost of all the Cooking oil consumed yesterday?
*Enter 999 if does not know*

p. Yesterday, did your household consume Tomatoes?

- Yes
- No

What do you estimate was the APPROXIMATE total cost of all the Tomatoes consumed yesterday?
*Enter 999 if does not know*

q. Yesterday, did your household consume Onions?

- Yes
- No

What do you estimate was the APPROXIMATE total cost of all the Onions consumed yesterday?
*Enter 999 if does not know*

r. In the last 7 days, did your household consume rape?

- Yes
- No

What do you estimate was the APPROXIMATE total cost of all the rape consumed in the last 7 days?
*Enter 999 if does not know*

t. In the last 7 days, did your household consume Cabbage?

- Yes
- No

What do you estimate was the APPROXIMATE total cost of all the Cabbage consumed in the last 7 days?
*Enter 999 if does not know*

Does this include the cabbage that was bought or produced earlier but was consumed in the last 7 days? **If not, go back and adjust the amount**

**C: HEALTH EXPENDITURES**

Now I would like to ask some questions about health care expenditures.

Q15 Has any member of your household visited a healthcare facility or provider or traditional healer to receive healthcare without staying overnight over the past 4 weeks?

- Yes
- No
- I don’t remember

Q16 How many members of your household visited the healthcare facility or provider or traditional healer in the past 4 weeks?

*Should be greater than 0 if yes to q 15*

**I am now going to ask you some questions about health care visits for household members in the past 4 weeks**

Q17a What was the total cost of the health care visits, including drugs, consultations, tests and supplies (including gloves), for all household members in the past 4 weeks? Do not include transport costs.
*Enter 999 if does not know*

Q17b What was the total transport cost to and from the facility/provider for health care visits for all household members in the past 4 weeks?
*Enter 999 if does not know*

Q18 In the **LAST 12 MONTHS,** was any member of your household admitted overnight to a healthcare facility?

- Yes
- No
- I don’t remember

Q19 How many members were admitted to a hospital in the last 12 months?

*Should be greater than 0 if yes to q 18*

**I am now going to ask you some questions about hospital admissions for household members in the past 12 months. I will ask questions separately for each household member that was admitted to hospital.**

**List admission costs hhm1**

Q20ia. What do you estimate the total cost of all hospital admissions of **household member 1** to have been in terms of hospital bills, drugs, supplies (syringes, gloves etc) in the past 12 months? Do not include transport costs.
*Enter 999 if does not know*

*If the respondent does not know the total cost immediately, fill in the fields below instead for the separate components*

Book

Drugs

Gloves

Other supplies

X-ray

Lab tests

Q20ib.What do you estimate the transport costs to and from the facility were for household members visiting the admitted **household member 1** during the hospital admissions in the past 12 months?
*Enter 999 if does not know*

Q20ic. What do you estimate the costs of food and drinks for the patient and accompanying member to have been during the hospital admissions for the admitted **household member 1** and accompanying care-taker members in the past 12 months?
*Enter 999 if does not know*

**List admission costs hhm2**

Q20iia. What do you estimate the total cost of all hospital admissions of **household member 2** to have been in terms of hospital bills, drugs, supplies (syringes, gloves etc) in the past 12 months? Do not include transport costs.
*Enter 999 if does not know*

*If the respondent does not know the total cost immediately, fill in the fields below instead for the separate components*

Book

Drugs

Gloves

Other supplies

X-ray

Lab tests

Q20iib.What do you estimate the transport costs to and from the facility were for household members visiting the admitted **household member 2** during the hospital admissions in the past 12 months?
*Enter 999 if does not know*

Q20iic. What do you estimate the costs of food and drinks for the patient and accompanying member to have been during the hospital admissions for the admitted **household member 2** and accompanying care-taker members in the past 12 months?
*Enter 999 if does not know*

**List admission costs hhm3**

Q20iiia. What do you estimate the total cost of all hospital admissions of **household member 3** to have been in terms of hospital bills, drugs, supplies (syringes, gloves etc) in the past 12 months? Do not include transport costs.
*Enter 999 if does not know*

*If the respondent does not know the total cost immediately, fill in the fields below instead for the separate components*

Book

Drugs

Gloves

Other supplies

X-ray

Lab tests

Q20iiib.What do you estimate the transport costs to and from the facility were for household members visiting the admitted **household member 3** during the hospital admissions in the past 12 months?
*Enter 999 if does not know*

Q20iiic. What do you estimate the costs of food and drinks for the patient and accompanying member to have been during the hospital admissions for the admitted **household member 3** and accompanying care-taker members in the past 12 months?
*Enter 999 if does not know*

**List admission costs hhm4**

Q20iva. What do you estimate the total cost of all hospital admissions of **household member 4** to have been in terms of hospital bills, drugs, supplies (syringes, gloves etc) in the past 12 months? Do not include transport costs.
*Enter 999 if does not know*

*If the respondent does not know the total cost immediately, fill in the fields below instead for the separate components*

Book

Drugs

Gloves

Other supplies

X-ray

Lab tests

Q20ivb.What do you estimate the transport costs to and from the facility were for household members visiting the admitted **household member 4** during the hospital admissions in the past 12 months?
*Enter 999 if does not know*

Q20ivc. What do you estimate the costs of food and drinks for the patient and accompanying member to have been during the hospital admissions for the admitted **household member 4** and accompanying care-taker members in the past 12 months?
*Enter 999 if does not know*

**List admission costs hhm5**

Q20va. What do you estimate the total cost of all hospital admissions of **household member 5** to have been in terms of hospital bills, drugs, supplies (syringes, gloves etc) in the past 12 months? Do not include transport costs.
*Enter 999 if does not know*

*If the respondent does not know the total cost immediately, fill in the fields below instead for the separate components*

Book

Drugs

Gloves

Other supplies

X-ray

Lab tests

Q20vb.What do you estimate the transport costs to and from the facility were for household members visiting the admitted **household member 5** during the hospital admissions in the past 12 months?

*Enter 999 if does not know*

Q20vc. What do you estimate the costs of food and drinks for the patient and accompanying member to have been during the hospital admissions for the admitted **household member 5** and accompanying care-taker members in the past 12 months?
*Enter 999 if does not know*

**List admission costs hhm6**

Q20via. What do you estimate the total cost of all hospital admissions of **household member 6** to have been in terms of hospital bills, drugs, supplies (syringes, gloves etc) in the past 12 months? Do not include transport costs.
*Enter 999 if does not know*

*If the respondent does not know the total cost immediately, fill in the fields below instead for the separate components*

Book

Drugs

Gloves

Other supplies

X-ray

Lab tests

Q20vib.What do you estimate the transport costs to and from the facility were for household members visiting the admitted **household member 6** during the hospital admissions in the past 12 months?
*Enter 999 if does not know*

Q20vic. What do you estimate the costs of food and drinks for the patient and accompanying member to have been during the hospital admissions for the admitted **household member 6** and accompanying care-taker members in the past 12 months?
*Enter 999 if does not know*

**List admission costs hhm7**

Q20viia. What do you estimate the total cost of all hospital admissions of **household member 7** to have been in terms of hospital bills, drugs, supplies (syringes, gloves etc) in the past 12 months? Do not include transport costs.*Enter 999 if does not know*

*If the respondent does not know the total cost immediately, fill in the fields below instead for the separate components*

Book

Drugs

Gloves

Other supplies

X-ray

Lab tests

Q20viib.What do you estimate the transport costs to and from the facility were for household members visiting the admitted **household member 7** during the hospital admissions in the past 12 months?
*Enter 999 if does not know*

Q20viic. What do you estimate the costs of food and drinks for the patient and accompanying member to have been during the hospital admissions for the admitted **household member 7** and accompanying care-taker members in the past 12 months?
*Enter 999 if does not know*

**List admission costs hhm8**

Q20viiia. What do you estimate the total cost of all hospital admissions of **household member 8** to have been in terms of hospital bills, drugs, supplies (syringes, gloves etc) in the past 12 months? Do not include transport costs.*Enter 999 if does not know*

*If the respondent does not know the total cost immediately, fill in the fields below instead for the separate components*

Book

Drugs

Gloves

Other supplies

X-ray

Lab tests

Q20viiib.What do you estimate the transport costs to and from the facility were for household members visiting the admitted **household member 8** during the hospital admissions in the past 12 months?
*Enter 999 if does not know*

Q20viiic. What do you estimate the costs of food and drinks for the patient and accompanying member to have been during the hospital admissions for the admitted **household member 8** and accompanying care-taker members in the past 12 months?
*Enter 999 if does not know*

**D: EXPENDITURES ON EDUCATION**

**Now I have some questions about expenditures related to education.**

Q21 How many members of your household are currently attending school (including the RISE participant)?

**Expenditures on education child one**

Q22ai. How much did you pay last year for tuition fees for the first child?
*Enter 999 if does not know*

Q22bi. How much did you pay last year for examination fees for the first child?
*Enter 999 if does not know*

Q22ci. How much did you pay last year for Uniforms for the first child?
*Enter 999 if does not know*

Q22di. How much did you pay last year for Stationeries for the first child?
*Enter 999 if does not know*

Q22ei. How much did you pay last year for Transport to go to school for the first child?
*Enter 999 if does not know*

Q22fi. How much did you pay last year for Boarding/other costs for the first child?
*Enter 999 if does not know*

**Expenditures on education child two**

Q22aii. How much did you pay last year for tuition fees for the second child?
*Enter 999 if does not know*

Q22bii. How much did you pay last year for examination fees for the second child?
*Enter 999 if does not know*

Q22cii. How much did you pay last year for Uniforms for the second child?
*Enter 999 if does not know*

Q22dii. How much did you pay last year for Stationeries for the second child?
*Enter 999 if does not know*

Q22eii. How much did you pay last year for Transport to go to school for the second child?
*Enter 999 if does not know*

Q22fii. How much did you pay last year for Boarding/other costs for the second child?
*Enter 999 if does not know*

**Expenditures on education child three**

Q22aiii. How much did you pay last year for tuition fees for the third child?
*Enter 999 if does not know*

Q22biii. How much did you pay last year for examination fees for the third child?
*Enter 999 if does not know*

Q22ciii. How much did you pay last year for Uniforms for the third child?
*Enter 999 if does not know*

Q22diii. How much did you pay last year for Stationeries for the third child?
*Enter 999 if does not know*

Q22eiii. How much did you pay last year for Transport to go to school for the third child?
*Enter 999 if does not know*

Q22fiii. How much did you pay last year for Boarding/other costs for the third child?
*Enter 999 if does not know*

**Expenditures on education child four**

Q22aiv. How much did you pay last year for tuition fees for the fourth child?
*Enter 999 if does not know*

Q22biv. How much did you pay last year for examination fees for the fourth child?
*Enter 999 if does not know*

Q22civ. How much did you pay last year for Uniforms for the fourth child?
*Enter 999 if does not know*

Q22div. How much did you pay last year for Stationeries for the fourth child?
*Enter 999 if does not know*

Q22eiv. How much did you pay last year for Transport to go to school for the fourth child?
*Enter 999 if does not know*

Q22fiv. How much did you pay last year for Boarding/other costs for the fourth child?
*Enter 999 if does not know*

**Expenditures on education child five**

Q22av. How much did you pay last year for tuition fees for the fifth child?
*Enter 999 if does not know*

Q22bv. How much did you pay last year for examination fees for the fifth child?
*Enter 999 if does not know*

Q22cv. How much did you pay last year for Uniforms for the fifth child?
*Enter 999 if does not know*

Q22dv. How much did you pay last year for Stationeries for the fifth child?
*Enter 999 if does not know*

Q22ev. How much did you pay last year for Transport for the to go to school fifth child?
*Enter 999 if does not know*

Q22fv. How much did you pay last year for Boarding/other costs for the fifth child?
*Enter 999 if does not know*

**Expenditures on education child six**

Q22avi. How much did you pay last year for tuition fees for the sixth child?
*Enter 999 if does not know*

Q22bvi. How much did you pay last year for examination fees for the sixth child?
*Enter 999 if does not know*

Q22cvi. How much did you pay last year for Uniforms for the sixth child?
*Enter 999 if does not know*

Q22dvi. How much did you pay last year for Stationeries for the sixth child?
*Enter 999 if does not know*

Q22evi. How much did you pay last year for Transport to go to school for the sixth child?
*Enter 999 if does not know*

Q22fvi. How much did you pay last year for Boarding/other costs for the sixth child?
*Enter 999 if does not know*

**Expenditures on education child seven**

Q22avii. How much did you pay last year for tuition fees for the seventh child?
*Enter 999 if does not know*

Q22bvii. How much did you pay last year for examination fees for the seventh child?
*Enter 999 if does not know*

Q22cvii. How much did you pay last year for Uniforms for the seventh child?
*Enter 999 if does not know*

Q22dvii. How much did you pay last year for Stationeries for the seventh child?
*Enter 999 if does not know*

Q22evii. How much did you pay last year for Transport to go to school for the seventh child?
*Enter 999 if does not know*

Q22fvii. How much did you pay last year for Boarding/ other costs for the seventh child?
*Enter 999 if does not know*

**Expenditures on education child eight**

Q22aviii. How much did you pay last year for tuition fees for the eighth child?
*Enter 999 if does not know*

Q22bviii. How much did you pay last year for examination fees for the eighth child?
*Enter 999 if does not know*

Q22cviii. How much did you pay last year for Uniforms for the eighth child?
*Enter 999 if does not know*

Q22dviii. How much did you pay last year for Stationeries for the eighth child?
*Enter 999 if does not know*

Q22eviii. How much did you pay last year for Transport to go to school for the eighth child?
*Enter 999 if does not know*

Q22fviii. How much did you pay last year for Boarding/other costs for the eighth child?
*Enter 999 if does not know*

**Expenditures on education child nine**

Q22aix. How much did you pay last year for tuition fees for the ninth child?
*Enter 999 if does not know*

Q22bix. How much did you pay last year for examination fees for the ninth child?
*Enter 999 if does not know*

Q22cix How much did you pay last year for Uniforms for the ninth child?
*Enter 999 if does not know*

Q22dix. How much did you pay last year for Stationeries for the ninth child?
*Enter 999 if does not know*

Q22eix. How much did you pay last year for Transport to go to school for the ninth child?
*Enter 999 if does not know*

Q22fix. How much did you pay last year for Boarding/ other costs for the ninth child?
*Enter 999 if does not know*

**Expenditures on education child ten**

Q22ax. How much did you pay last year for tuition fees for the tenth child?
*Enter 999 if does not know*

Q22bx. How much did you pay last year for examination fees for the tenth child?
*Enter 999 if does not know*

Q22cx. How much did you pay last year for Uniforms for the tenth child?
*Enter 999 if does not know*

Q22dx. How much did you pay last year for Stationeries for the tenth child?
*Enter 999 if does not know*

Q22ex. How much did you pay last year for Transport to go to school for the tenth child?
*Enter 999 if does not know*

Q22fx. How much did you pay last year for Boarding/other costs for the tenth child?
*Enter 999 if does not know*

**Expenditures on education child eleven**

Q22axi. How much did you pay last year for tuition fees for the eleventh child?
*Enter 999 if does not know*

Q22bxi. How much did you pay last year for examination fees for the eleventh child?
*Enter 999 if does not know*

Q22cxi. How much did you pay last year for Uniforms for the eleventh child?
*Enter 999 if does not know*

Q22dxi. How much did you pay last year for Stationeries for the eleventh child?
*Enter 999 if does not know*

Q22exi. How much did you pay last year for Transport to go to school for the eleventh child?
*Enter 999 if does not know*

Q22fxi. How much did you pay last year for Boarding/other costs for the eleventh child?
*Enter 999 if does not know*

**Expenditures on education child twelve**

Q22axii. How much did you pay last year for tuition fees for the twelveth child?
*Enter 999 if does not know*

Q22bxii. How much did you pay last year for examination fees for the twelveth child?
*Enter 999 if does not know*

Q22cxii. How much did you pay last year for Uniforms for the twelveth child?
*Enter 999 if does not know*

Q22dxii. How much did you pay last year for Stationeries for the twelveth child?
*Enter 999 if does not know*

Q22exii. How much did you pay last year for Transport to go to school for the twelveth child?
*Enter 999 if does not know*

Q22fxii. How much did you pay last year for Boarding/other costs for the twelveth child?
*Enter 999 if does not know*

**Expenditures on education child thirteen**

Q22axiii. How much did you pay last year for tuition fees for the thirteenth child?
*Enter 999 if does not know*

Q22bxiii. How much did you pay last year for examination fees for the thirteenth child?
*Enter 999 if does not know*

Q22cxiii. How much did you pay last year for Uniforms for the thirteenth child?
*Enter 999 if does not know*

Q22dxiii. How much did you pay last year for Stationeries for the thirteenth child?
*Enter 999 if does not know*

Q22exiii. How much did you pay last year for Transport to go to school for the thirteenth child?
*Enter 999 if does not know*

Q22fxiii. How much did you pay last year for Boarding/other costs for the thirteenth child?
*Enter 999 if does not know*

**Expenditures on education child fourteen**

Q22axiv. How much did you pay last year for tuition fees for the fourteenth child?
*Enter 999 if does not know*

Q22bxiv. How much did you pay last year for examination fees for the fourteenth child?
*Enter 999 if does not know*

Q22cxiv. How much did you pay last year for Uniforms for the fourteenth child?
*Enter 999 if does not know*

Q22dxiv. How much did you pay last year for Stationeries for the fourteenth child?
*Enter 999 if does not know*

Q22exiv. How much did you pay last year for Transport to go to school for the fourteenth child?
*Enter 999 if does not know*

Q22fxiv. How much did you pay last year for Boarding/other costs for the fourteenth child?
*Enter 999 if does not know*

**Expenditures on education child fifteen**

Q22axv. How much did you pay last year for tuition fees for the fifteenth child?
*Enter 999 if does not know*

Q22bxv. How much did you pay last year for examination fees for the fifteenth child?
*Enter 999 if does not know*

Q22cxv. How much did you pay last year for Uniforms for the fifteenth child?
*Enter 999 if does not know*

Q22dxv. How much did you pay last year for Stationeries for the fifteenth child?
*Enter 999 if does not know*

Q22exv. How much did you pay last year for Transport to go to school for the fifteenth child?
*Enter 999 if does not know*

Q22fxv. How much did you pay last year for Boarding/other costs for the fifteenth child?
*Enter 999 if does not know*

**E: OTHER EXPENDITURES**

I would also like to find out how much you spend on other household expenses.

Q23 How much does your household spend on any of the following items per month?

Item purchased or paid

Q23iia. Do you purchase soap?

- Yes
- No

Q23iie. What is the estimated cost of soap in a month?
*Enter 999 if does not know*

Q23iiia. Do you purchase personal care items (tooth paste, lotion etc)?

- Yes
- No

Q23iiie. What is the estimated cost of personal care items (tooth paste, lotion etc) in a month ?
*Enter 999 if does not know*

Q23va. Do you purchase Electricity?

- Yes
- No

Q23ve. What is the estimated cost of Electricity in a month?
*Enter 999 if does not know*

Q23viia. Do you purchase Petrol/diesel?

- Yes
- No

Q23viie. What is the estimated cost of Petrol/diesel in a month?
*Enter 999 if does not know*

Q23viiia. Do you purchase Charcoal?

- Yes
- No

Q23viiie. What is the estimated cost of Charcoal in a month?
*Enter 999 if does not know*

Q23ixa. Do you purchase Batteries?

- Yes
- No

Q23ixe. What is the estimated cost of Batteries in a month?
*Enter 999 if does not know*

Q23xa. Do you pay a salary to a permanent employee (for example a maid or a cattle herder)?

- Yes
- No

Q23xe. How much did you spend on salaries to permanent employees last month?
*Enter 999 if does not know*

Q23xei. Other modes of payment (other than money).

Q23xia. Do you pay a salary to a seasonal worker or someone doing piecework?

- Yes
- No

Q23xib. How many months in a year do you pay seasonal workers and those doing piecework?
*Enter 999 if does not know*

Q23xie. How much did you spend on salaries to seasonal workers and those doing piecework last month?
*Enter 999 if does not know*

Q23xia. Do you pay for Public transport?

- Yes
- No

Q23xie. What is the estimated cost of public transport in a month ?
*Enter 999 if does not know*

Q23xiiia. Do you purchase air time?

- Yes
- No

Q23xie. What is the estimated cost of airtime in a month?
*Enter 999 if does not know*

Q24 On average, how much did your household spend to buy or maintain the following items in the past 1 year?

Q24ia. Did you or anyone else in the household purchase or repair any Shoes or clothes in the past 1 year?

- Yes
- No

Q24ic. What do you estimate the total cost of purchasing and/or repairing shoes or clothes in the past 1 year to have been?
*Enter 999 if does not know*

Q24iva. Did you buy Fertilizers or seeds in the past 1 year?

- Yes
- No

Q24ivc. What do you estimate the total cost of purchasing Fertilizers and seeds in the past 1 year to have been?
*Enter 999 if does not know*

Q24viia. Did you or anyone else in the household purchase or repair any Kitchen utensils (used for cooking and eating) in the past 1 year?

- Yes
- No

Q24viic. What do you estimate the total cost of purchasing and/or repairing kitchen utensils in the past 1 year to have been?
*Enter 999 if does not know*

Q24viiia. Did you or anyone else in the household purchase or repair any radio in the past 1 year?

- Yes
- No

Q24viiic. What do you estimate the total cost of purchasing and/or repairing a Radio in the past 1 year to have been?
*Enter 999 if does not know*

Q24xia. Did you or anyone else in the household purchase or repair any Cell phones in the past 1 year?

- Yes
- No

Q24xic. What do you estimate the total cost of purchasing and/or repairing Cell phones in the past 1 year to have been?
*Enter 999 if does not know*

Q24xiiia. Did you you or anyone else in the household purchase or repair any ox carts in the past 1 year?

- Yes
- No

Q24xiiic. What do you estimate the total cost of purchasing and/or repairing ox carts in the past 1 year to have been?
*Enter 999 if does not know*

Q24xiva. Did you or anyone else in the household purchase or repair any bicycles in the past 1 year?

- Yes
- No

Q24xivc. What do you estimate the total cost of purchasing and/or repairing bicycles in the past 1 year to have been?
*Enter 999 if does not know*

Q24xvia. Did you or anyone else in the household purchase or repair any cars in the past 1 year?

- Yes
- No

Q24xvic. What do you estimate the total cost of purchasing and/or repairing cars in the past 1 year to have been?
*Enter 999 if does not know*

Q24xviia. Did you or anyone else in the household buy any building materials in the past 1 year?

- Yes
- No

Q24xviic. What do you estimate the total cost of purchasing building materials in the past 1 year to have been?
*Enter 999 if does not know*

Q24xviiia. Did you spend money on maintaining a borehole in the past 1 year?

- Yes
- No

Q24xviiic. What do you estimate the total cost of maintaining the borehole in the past 1 year to have been?
*Enter 999 if does not know*

**F: ATTITUDES**

**We are now moving to the second part of the questionnaire where we will ask questions about your attitudes regarding schooling, contraceptives, marriage and childbearing.
I will ask you some questions about your opinion regarding schooling. I am interested in your opinion, and no answers are more correct than others. I am going to read to you a series of statements regarding girls and schooling, sexual issues, marriage and childbearing.**

Q25 Do you think girls have the same chances as boys to find a job if they finish grade 9.

- Yes
- No
- Don't Know

Q26 Do you think <output value="/data/preloads_group/prev_girl_name"/> is ready to learn about sexual and reproductive issues (abstinence, controlling sexual desires, condoms, contraceptives).

- Yes
- No
- Don't Know

Q27 Do you feel comfortable talking to <output value="/data/preloads_group/prev_girl_name"/> about romantic relationships and sexual issues?

- Yes
- No
- Don't Know

Q28 Do you think a grade 8 or 9 girl who is sexually active but not married should be encouraged to use contraceptives, for example injections/pills or condoms.

- Yes
- No
- Don't Know

Q29 Do you think an out-of-school girl who has reached puberty and who is sexually active but not married should be encouraged to use contraceptives, for example injections/pills, condoms.

- Yes
- No
- Don't Know

Q30 Do you think it is harmful for a girl who has reached puberty and who is sexually active but not married to use contraceptives, for example injections/pills.

- Yes
- No
- Don't Know

**Now I have some questions regarding your opinions about when girls should get married.**

Q31a. What do you think are the three most important characteristics of a girl who should be encouraged to get married?

*Tick the first three responses. If they only give 1 or 2 responses, ask for more characteristics.*

- out-of-school
- pregnant or having a child
- age
- stature like a grown woman
- reached puberty
- economically vulnerable
- economically stable
- orphan
- other

Q31b. Other, specify

Q32 In your opinion, what is the best age for a girl to get married?

Q33 Do you think a girl who has reached puberty and who is out of school should be encouraged to get married

- Yes
- No
- Don't Know

**Then I would like to get your opinion on when it is good for a young girl to have a child.**

34a. What do you think are the three most important characteristics of a girl who can benefit from having a child? The benefits could be increased respect or social status.

*Tick the first three responses. If they only give 1 or 2 responses, ask for more characteristics.*

- out-of-school
- married
- age
- stature like a grown woman
- reached puberty
- economically vulnerable
- economically stable
- orphan
- other

Q34b. Other, specify

Q35 Do you think unmarried girls who have reached puberty and who have a child are treated with more respect than girls of the same age who do not have a child.

- Yes
- No
- Don't Know

Q36 Do you think unmarried girls who have reached puberty and who have a child are a significant economic burden to their family.

- Yes
- No
- Don't Know

**I will now ask you questions about health insurance/medical scheme.
Health insurance (Medical scheme) means a household contributes a certain amount of money for healthcare each month even when there is no one who is sick so that when any member gets sick he/she can receive free healthcare services simply by showing an insurance card without paying anything at the facility. It may help to protect the household from paying large amounts of money that at times may force the household to sell properties in the event of a serious illness.

In relation to this, I have some questions about health insurance.**

Q37a. Does your household currently make monthly contributions to a health insurance/medical scheme?

- Yes
- No
- Don't Know

Q37b. How much do you pay per year for the medical scheme?
*Enter 999 if does not know*

Q38. If you were to contribute a certain amount of money for health insurance each month to ensure that all members of your household receive free healthcare services, how much would you be willing to contribute per month?
*If the respondent does not know, enter 99*

Q39 Have you or any member of your household ever attended any RISE meetings?

- Yes
- No
- I don’t know

Q40 How many RISE meetings have you attended in the last 6 months?

*If the respondent does not remember, enter 99*

list phone numbers

Which phone number may we use if we want to get in touch with you at a later point in time?

Number 1 ___________________

Number 2___________________

Number 3___________________

Which phone number can be used to contact xx if we are not able to find her the next time we come to conduct an interview? ___________________

Is this guardian representing other girls also? List the names here___________________

Thanks a lot for participating in this interview!

To the research assistant: *Are there any inconsistencies in what the parent/ guardian has reported that you would like to comment on?*

Take a picture together with the parent and ask her/him to sign and put a thumb print on your list to verify that you have interviewed her/him.

Capture location_____
