# Supplementary material for: Parent-child communication about sexual issues in Zambia: a cross sectional study of adolescent girls and their parents
Source: BMC Public Health. 2020 Jul 16;20:1120. doi: 10.1186/s12889-020-09218-y (PMC7364553; doi:10.1186/s12889-020-09218-y)
Supplement: Supplementary file 2 — Additional file 2 Additional data on distribution of responses for occurrence of parent-child communication about sexual issues and psychosocial variables used in the study. Table 1 presents the distribution of responses to ‘Occurrence of parent-child communication about sexual issues’ broken down by the five original response categories. This shows the distribution of responses before the variable was converted to a dichotomous variable. Table 2 shows the response patterns for the psychosocial variables according to the five original response options for the frequency of parent-child sexuality communication (“How many times have you talked about romantic relationships or sexual issues.”). [file 12889_2020_9218_MOESM2_ESM.docx]

## Additional File 2

## Additional data on distribution of responses for occurrence of parent-child communication about sexual issues and psychosocial variables used in the study.

Table 1. Distribution of responses to ‘*Occurrence of parent-child communication about sexual issues’* broken down by the five original response categories (before conversion to dichotomous variable, as used in the final analysis)

| Frequency | N(%) |
| --- | --- |
| 5 or more times | 751 (17%) |
| 2-4 times | 485 (11%) |
| Once | 285 (7%) |
| Never | 2713 (62%) |
| I don’t Know | 109 (3%) |

Table 2. Response patterns for independent/psychosocial variables according to the five original response options for the frequency of parent-child sexuality communication (“*How many times have you talked about romantic relationships or sexual issues*.”).

|  | Ever communicated about sex | | | |  |
| --- | --- | --- | --- | --- | --- |
|  | 5 or more | 2-4 times | 1 time | Never | Don’t know |
| *Girl’s connectedness* |  |  |  |  |  |
| Yes | 574 (77%) | 384 (79%) | 224 (79%) | 2038 (75%) | 81 (76%) |
| No | 137 (18%) | 90 (19%) | 57 (20%) | 613 (23%) | 19 (18%) |
| Don’t Know | 37 (5%) | 10 (2%) | 4 (1%) | 65 (2%0 | 7 (7%) |
| *Girl-reported fear-based communication* |  |  |  |  |  |
| Yes | 331 (44%) | 217 (45%) | 115 (40%) | 1371 (51%) | 56 (52%) |
| No | 395 (53%) | 260 (54%) | 164 (58%) | 1242 (46%) | 41 (38%) |
| Don’t Know | 22 (3%) | 7 (1%) | 6 (2%) | 94 (3%) | 10 (9%) |
| *Girl’s perceived parental comfort* |  |  |  |  |  |
| Yes | 546 (73%) | 276 (57%) | 141 (49%) | 935 (36%) | 55 (51%) |
| No | 197 (26%) | 198 (41%) | 139 (49%) | 1721 (64%) | 46 (43%) |
| Don’t Know | 5 (0.7%) | 10 (2%) | 5 (2%) | 50 (2%) | 6 (6%) |
| *Girl perceives parent as objecting to contraception education* |  |  |  |  |  |
| Yes | 310 (41%) | 225 (46%) | 132 (46%) | 1442 (53%) | 45 (42%) |
| No | 415 (55%) | 244 (50%) | 146 (51%) | 1194 (44%) | 57 (53%) |
| Don’t Know | 23 (3%) | 15 (3%) | 7 (2%) | 71 (3%) | 5 (5%) |
| PARENTAL VARIABLES | | | |  |  |
| *Parent’s comfort* |  |  |  |  |  |
| Yes | 479 (77%) | 308 (74%) | 173 (73%) | 1671 (76%) | 70 (79%) |
| No | 140 (23%) | 106 (25%) | 63 (27%) | 527 (24%) | 19 (21%) |
| Don’t Know | 3 (0.5%) | 2 (0.5%) | 0 | 9 (0.4%) | 0 |
| *Parent’s daughter ready for SRH education* |  |  |  |  |  |
| Yes | 376 (60%) | 250 (60%) | 120 (51%) | 1229 (56%) | 47 (53%) |
| No | 229 (37%) | 152 (37%) | 105 (44%) | 902 (41%) | 40 (45%) |
| Don’t Know | 18 (3%) | 14 (3%) | 11 (5%) | 77 (3%) | 2 (2%) |
| *Parent’s perception of contraceptive harm* |  |  |  |  |  |
| Yes | 374 (60%) | 242 (58%) | 137 (58%) | 1320 (60%) | 47 (53%) |
| No | 233 (37%) | 162 (39%) | 93 (39%) | 828 (38%) | 41 (46%) |
| Don’t Know | 15 (2%) | 12 (3%) | 6 (3%) | 54 (2%) | 1 (1%) |
