# Supplementary material for: Parent-child communication about sexual issues in Zambia: a cross sectional study of adolescent girls and their parents
Source: BMC Public Health. 2020 Jul 16;20:1120. doi: 10.1186/s12889-020-09218-y (PMC7364553; doi:10.1186/s12889-020-09218-y)
Supplement: Supplementary file 3 — Additional file 3. Comparison of Model 3 results (sociodemographic and psychosocial variables) between the three groups in the RISE study (Control, Economic intervention, Combined intervention) and the pooled data. The table shows the results of the multivariate regression for Model 3 – which included both the psychosocial and sociodemographic variables – when the data was disaggregated according to the three groups in the RISE study: the control group, the economic intervention and the combined intervention group. OR and AORs are included for each of the variables in each of the groups. For comparison, the regression results for the pooled data (as presented in the paper) are also included/repeated in the table. [file 12889_2020_9218_MOESM3_ESM.docx]

## Additional File 3

## Comparison of Model 3 results (sociodemographic and psychosocial variables) between the intervention groups in the RISE study (Control, Economic intervention, Combined intervention) and the pooled data.

|  | Control (1)  N= 968 | | Economic Intervention (2)  N= 1978 | | Combined Intervention (3)  N=1887 | | Pooled Sample (as in paper)  N=4333 | |
| --- | --- | --- | --- | --- | --- | --- | --- | --- |
| **SOCIODEMOGRAPHIC VARIABLES** | OR | AOR | OR | AOR | OR | AOR | OR | AOR |
|  | |  |  |  |  |  |  | |
| *Girl age* | 1.16 (1.07-1.27) | 1.06 (0.93-1.21) | 1.16 (1.09-1.23) | 1.13 (1.05-1.21) | 0.96 (0.89-1.04) | 0.90 (0.82-1.00) | 1.08 (1.03-1.13) | 1.03 (0.97-1.09) |
| *Girl in school* |  |  |  |  |  |  |  | |
| Yes | 0.46 (0.31-0.69) | 0.62 (0.36-1.06) | 0.49 (0.35-0.6) | 0.52 (0.36-0.73) | 0.68 (0.50-0.94) | 0.38 (0.26-0.58) | 0.55 (0.45-0.67) | 0.54 (0.42-0.69) |
| No | Ref. | Ref. | Ref. | Ref. | Ref. | Ref. | Ref. | |
| *Sex of parent* |  |  |  |  |  |  |  | |
| Female | 1.02(0.68-1.53) | 1.00(0.66-1.52) | 0.82(0.62-1.07) | 0.86(0.65-1.13) | 1.19(0.95-1.48) | 1.32(1.05-1.66) | 0.99 (0.84-1.17) | 1.03 (0.87-1.22) |
| Male | Ref. | Ref. | Ref. | Ref. | Ref. | Ref. | Ref. | |
| *Parent’s education level* |  |  |  |  |  |  |  | |
| Primary or none | Ref. | Ref. | Ref. | Ref. | Ref. | Ref. | Ref. | |
| Secondary level | 0.95 (0.70-1.27) | 1.00 (0.73-1.36) | 1.14(0.91-1.44) | 1.21 (0.92-1.60) | 1.04 (0.83-1.29) | 1.04 (0.85-1.27) | 1.06 (0.92-1.22) | 1.09 (0.93-1.26) |
| Diploma or University | 0.33(0.13-0.87) | 0.26 (0.09-0.73) | 0.98(0.57-1.68) | 1.00 (0.59-1.70) | 0.75(0.49-1.13) | 0.61 (0.37-1.00) | 0.75 (0.55-1.03) | 0.71 (0.51-1.00) |
|  |  | |  | |  | |  | |
| **PSYCHOSOCIAL VARIABLES** |  |  |  |  |  |  |  | |
|  | |  |  |  |  |  |  | |
| *Girl’s connectedness* |  |  |  |  |  |  |  | |
| Yes | 1.2(0.84-1.70) | 1.25 (0.87-1.78) | 1.09(0.82-1.46) | 1.13 (0.77-1.64) | 1.46(1.09-1.94) | 1.46 (1.06-2.02) | 1.25 (1.04-1.49) | 1.26 (1.03-1.55) |
| No | Ref. | Ref. | Ref. | Ref. | Ref. | Ref. | Ref. | |
| Don’t Know | 1.80 (0.73-4.42) | 2.23 (1.05-4.76) | 1.79(0.83-3.85) | 1.89 (0.77-4.64) | 2.31(0.94-5.6) | 2.10 (0.77-5.70) | 1.96 (1.20-3.20) | 2.0 (1.21-3.41) |
| *Girl-reported fear-based communication* |  |  |  |  |  |  |  | |
| Yes | 0.89(0.68-1.17) | 0.93 (0.68-1.28) | 0.73(0.58-0.92) | 0.71 (0.54-0.92) | 0.67(0.54-0.82) | 0.64 (0.49-0.83) | 0.73 (0.64-0.83) | 0.72(0.61-0.85) |
| No | Ref. | Ref. | Ref. | Ref. | Ref. | Ref. | Ref. | |
| Don’t Know | 0.61 (0.23-1.56) | 0.78 (0.28-2.14) | 0.79(0.42-1.48) | 0.70 (0.30-1.66) | 0.35(0.17-0.70) | 0.44 (0.19-1.00) | 0.56 (0.37-0.85) | 0.63 (0.38-1.04) |
| *Girl’s perceived parental comfort* |  |  |  |  |  |  |  | |
| Yes | 3.35 (2.50-4.49) | 3.11 (2.17-4.46) | 2.94 (2.29-3.77) | 2.89 (2.17-3.85) | 3.74 (2.73-5.10) | 3.25 (2.31-4.56) | 3.31 (2.80-3.92) | 2.98 (2.47-3.60) |
| No | Ref. | Ref. | Ref. | Ref. | Ref. | Ref. | Ref. | |
| Don’t Know | 1.32 (0.33-5.20) | 1.20 (0.28-5.14) | 1.29 (0.46-3.61) | 1.07 (0.35-3.20) | 1.26 (0.49-3.24) | 0.85 (0.32- 2.27) | 1.28 (0.68-2.41) | 1.02 (0.55-1.89) |
| *Girl perceives parent as objecting to contraception education* |  |  |  |  |  |  |  | |
| Yes | 0.59 (0.41-0.86) | 0.61 (0.38-0.98) | 0.76 (0.60-0.97) | 0.87 (0.66-1.14) | 0.65 (0.50-0.84) | 0.81 (0.59-1.11) | 0.68 (0.58-0.80) | 0.77 (0.64-0.93) |
| No | Ref. | Ref. | Ref. | Ref. | Ref. | Ref. | Ref. | |
| Don’t Know | 1.06 (0.40-2.77) | 1.33 (0.41-4.25) | 0.61 (0.32-1.17) | 0.27 (0.11-0.65) | 1.35 (0.68-2.65) | 1.43 (0.65-3.16) | 0.94 (0.61-1.44) | 0.73 (0.42-1.27) |
|  |  | | | |  |  |  | |
| *Parent’s comfort* |  |  |  |  |  |  |  | |
| Yes | 1.53(1.04-2.25) | 1.45 (0.92-2.27) | 0.75 (0.58-0.96) | 0.69 (0.52-0.92) | 1.06(0.79-1.31) | 0.91 (0.67-1.23) | 0.97 (0.81-1.17) | 0.86 (0.70-1.05) |
| No | Ref. | Ref. | Ref. | Ref. | Ref. | Ref. | Ref. | |
| Don’t Know | 1 | 1 | 0.35 (0.03-3.52) | 0.31 (0.03-3.34) | 2.25(0.48-10.56) | 3.57 (0.73-17.30) | 0.94 (0.30-2.90) | 0.92 (0.27-3.16) |
| *Parent’s daughter ready for SRH education* |  |  |  |  |  |  |  | |
| Yes | 1.17 (0.84-1.62) | 1.09 (0.78-1.51) | 0.98 (0.76-1.26) | 0.92 (0.69-1.23) | 1.25 (0.96-1.64) | 1.23 (0.90-1.68) | 1.12 (0.96-1.31) | 1.07 (0.90-1.28) |
| No | Ref. | Ref. | Ref. | Ref. | Ref. | Ref. | Ref. | |
| Maybe | 1.27 (0.59-2.76) | 1.48 (0.62-3.51) | 1.12 (0.63-2.00) | 0.94 (0.47-1.90) | 0.82 (0.41-1.63) | 0.74 (0.35-1.55) | 1.03 (0.70-1.52) | 0.95 (0.62-1.45) |
| *Parent’s perception of contraceptive harm* |  |  |  |  |  |  |  | |
| Yes | 0.71 (0.47-0.16) | 0.75 (0.47-1.20 | 0.97 (0.78-1.21) | 1.00 (0.78-1.27) | 1.11 (0.88-1.39) | 1.09 (0.87-1.37) | 0.96 (0.83-1.12) | 0.98 (0.84-1.15) |
| No | Ref. | Ref. | Ref. | Ref. | Ref. | Ref. | Ref. | |
| Don’t Know | 0.41 (0.09-1.84) | 0.42(0.09-1.91) | 1.22 (0.57-2.62) | 1.02 (0.42-2.43) | 1.32 (0.72-2.44) | 1.08 (0.56-2.10) | 1.03 (0.64-1.67) | 0.91 (0.55-1.50) |
